# Clinical Features of Adult Patients with Isolated Pulmonary Valve Endocarditis: A Systematic Review

**DOI:** 10.3390/microorganisms14061208

**Published:** 2026-05-27

**Authors:** Guillermo Cuervo, Supavit Chesdachai, Joya-Rita Hindy, Danielle Gerberi, Christopher V. DeSimone, Abhishek J. Deshmukh, José M. Miró, Abdallah El Sabbagh, Daniel C. DeSimone, Larry M. Baddour

**Affiliations:** 1 Infectious Diseases Department, Hospital Clinic-IDIBAPS, University of Barcelona, 08036 Barcelona, Spain; josemaria@miromoreno.org; 2 CIBERINFEC, Instituto de Salud Carlos III, 28029 Madrid, Spain; 3 Department of Medicine, Division of Public Health, Infectious Diseases and Occupational Medicine, Mayo Clinic, Rochester, MN 55902, USA; chesdachai.supavit@mayo.edu (S.C.); hindy.joya-rita@mayo.edu (J.-R.H.); desimone.daniel@mayo.edu (D.C.D.);; 4 Department of Cardiovascular Medicine, Mayo Clinic, Rochester, MN 55902, USA; desimone.christopher@mayo.edu (C.V.D.); deshmukh.abhishek@mayo.edu (A.J.D.); 5 Department of Library Services, Mayo Clinic, Rochester, MN 55902, USA; gerberi.danielle@mayo.edu; 6 Reial Academia de Medicina de Catalunya, 08001 Barcelona, Spain; 7 Department of Cardiovascular Medicine, Mayo Clinic, Jacksonville, FL 32224, USA; elsabbagh.abdallah@mayo.edu

**Keywords:** infective endocarditis, pulmonic valve endocarditis, systematic review, microbiology, risk factors, outcomes, right-sided infective endocarditis

## Abstract

Isolated pulmonary valve endocarditis (PV-IE) is a rare condition. Its epidemiology, clinical profile, and management remain poorly characterized. A systematic review was conducted to provide a contemporary characterization (2020–2025) of adult cases with isolated PV-IE. Individuals with previous cardiac surgeries, cardiac implantable electronic devices (CIEDs), or long-term venous catheters were excluded. Of 1902 citations identified, 72 studies were selected with 79 eligible cases, mostly case reports. Patients were predominantly male (78.5%) with a median age of 43 years. Congenital heart disease (CHD) and injection drug use (IDU) were risk factors in 30.4% and 27.8% of cases, respectively, while 41.8% had neither (No-CHD/IDU group). *Staphylococcus aureus* was the leading pathogen (39%), followed by streptococci (16.5%) and *Enterococcus faecalis* (8.9%). Vegetations were large (median, 19.5 mm), and pulmonary septic emboli highly prevalent (~93% of cases). More than half (54.4%) of patients underwent surgery. Hospital mortality was low (7.6%). Differences across risk factor subgroups were observed, with higher median age in No-CHD/IDU patients and more staphylococcal etiology in patients with IDU, although treatment approaches and mortality did not differ. This review highlights distinct characteristics and the overall favorable short-term prognosis of isolated PV-IE, underscoring the need for larger, systematically collected datasets.

## 1. Introduction

Approximately 20% of all infective endocarditis (IE) cases involve the right side of the heart, often with cardiovascular implantable electronic devices (CIEDs), cardiac valves, or mural atrial and ventricular surfaces, usually associated with indwelling vascular catheters [1]. These factors are subject to significant geographical variations related to the epidemic of IDU that has greatly impacted some countries. Pulmonary valve endocarditis (PV-IE) is rare, especially as an isolated involvement, occurring in around 2% of IE cases in a single-center study [2] and 1.64% of IE cases (125 out of 7116) in the International Collaboration on Endocarditis, being more common in IDU patients [3].

Risk factors associated with PV-IE include the presence of long-term central venous catheters, CIED leads, congenital heart disease (CHD), and IDU [2,4,5,6]. Notable characteristics include diagnostic difficulties related to potential limitations of echocardiography in visualizing the pulmonary valve [6] and a low mortality rate [7,8]. However, the limited knowledge available is derived from isolated case reports or small case series. In this work, we conducted a systematic review of the literature with a focus on PV-IE prevalence, epidemiology, clinical profile, and its current diagnostic and therapeutic approach and prognosis according to contemporary publications.

## 2. Materials and Methods

We performed a systematic review of contemporary published cases/studies of adult (> or =18 years) patients diagnosed with isolated PV-IE between 2020 and November 2025. Studies without specific patient data or those published in languages other than English were excluded. Individuals were excluded if they had any other cardiac valve or cardiac structure involvement (e.g., tricuspid valve, Thebesian valve, Eustachian valve, Chiari network), prior cardiac surgeries, a cardiac implantable electronic device (CIED) in place, or central venous catheters (such as a hemodialysis catheter or another intravenous long-term catheter). Key definitions included: (a) definite infective endocarditis according to modified Duke or Duke-ISCVID criteria [9,10]; (b) congenital heart disease, as an abnormality in the heart’s structure or function (chambers, walls, valves, vessels) present at birth; and (c) IDU, considered as the self-administration of non-prescribed substances delivered by needle.

### Systematic Review

The medical literature was searched by an expert medical librarian (DG) for the concepts of non-tricuspid right-sided endocarditis. Search strategies were created using a combination of keywords and standardized index terms. Searches were run on 4 November 2025, in Ovid Cochrane Central Register of Controlled Trials (1991+), Ovid Embase (1974+, including records from ClinicalTrials.gov), Ovid Medline (1946+ including epub ahead of print, in-process, and other non-indexed citations), Scopus (1788+), and Web of Science Core Collection (Science Citation Index Expanded 1975+ and Emerging Sources Citation Index 2015+). After removing animal and pediatric studies and limiting to results from 2005+, a total of 2958 citations were retrieved. Deduplication was performed automatically in Covidence, leaving 1902 citations for screening. Full search strategies are provided in File S1 of the Supplementary Materials. Covidence systematic review software (Veritas Health Innovation, Melbourne, Australia. Available at www.covidence.org) was used to systematically manage the study selection process. Two independent reviewers (GC, LMB) evaluated titles and abstracts, as well as full texts, according to predefined inclusion and exclusion criteria. Discrepancies were resolved through consensus. Descriptive statistics were used and reported as median for continuous variables and count (percentage) for categorical variables. This systematic review was conducted and reported in accordance with the Preferred Reporting Items for Systematic Reviews and Meta-Analyses (PRISMA) 2020 guidelines (see PRISMA 2020 checklist on File S2 of the Supplementary Materials [11]).

## 3. Results

### 3.1. Systematic Review

**Figure 1:**
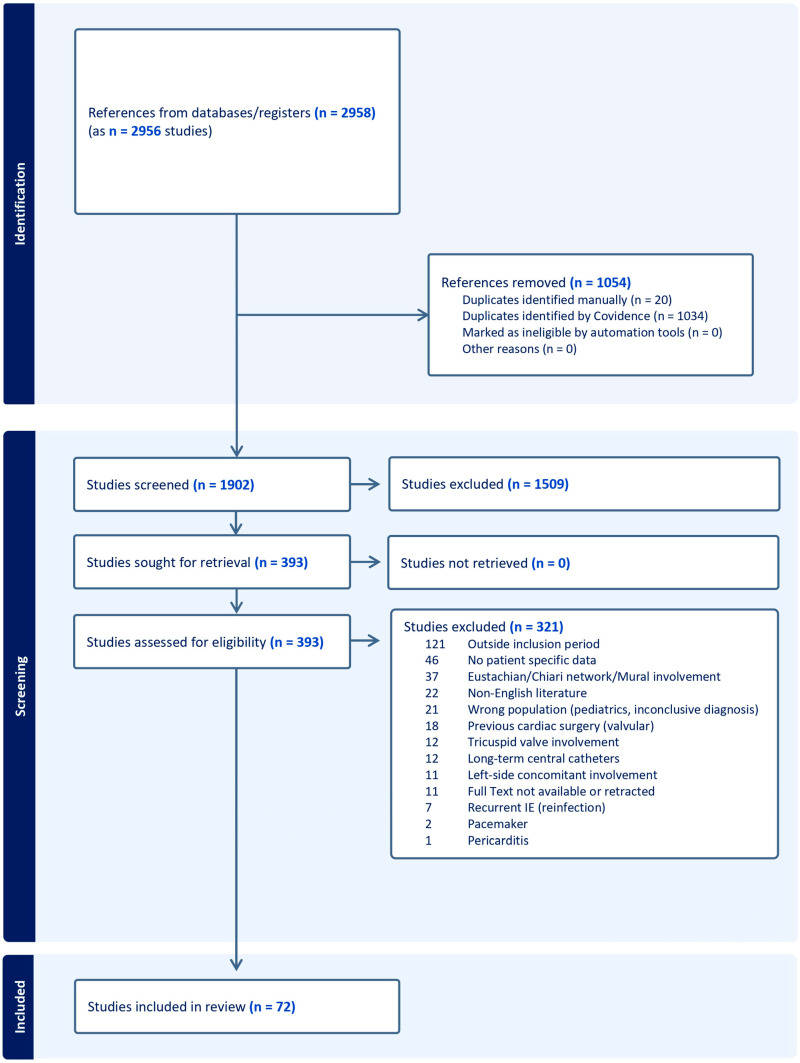
PRISMA flowchart: output of the systematic review.

The output of the systematic review is summarized in a flowchart (Figure 1). Briefly, 1902 references were identified, 265 full texts were reviewed, and ultimately, 72 citations with 79 cases that met inclusion criteria were compiled [5,6,7,12,13,14,15,16,17,18,19,20,21,22,23,24,25,26,27,28,29,30,31,32,33,34,35,36,37,38,39,40,41,42,43,44,45,46,47,48,49,50,51,52,53,54,55,56,57,58,59,60,61,62,63,64,65,66,67,68,69,70,71,72,73,74,75,76,77,78,79,80]. All except three articles were single case reports (27 of them were conference abstracts). The quality assessment of the included articles was carried out using the Joanna Briggs Institute Critical Appraisal Checklist for Case Reports [81] and is summarized in File S3 of the Supplementary Materials.

### 3.2. Baseline Demographics

Pooled analysis of the 79 patients showed that they were largely male (n = 62/78.5%) with a median age of 43 years (IQR: 32–57 years). Regarding associated risk factors, 24 patients (30.4%) had CHD, 22 (27.8%) had a history of IDU, and 33 (41.8%) of them had neither CHD nor IDU (No-CHD/IDU group).

### 3.3. Clinical and Echocardiographic Characteristics

Table 1 summarizes the microbiology, clinical characteristics, and imaging findings. The predominant pathogen was *Staphylococcus aureus* (n = 31/39%), with MSSA in 20 cases (25.3%) and MRSA in 11 (13.9%). Thirteen (16.5%) patients had streptococcal IE, and seven (8.9%) had *Enterococcus faecalis* IE. Blood-culture-negative IE occurred in 11 (13.9%) cases, and 17 (21.5%) cases were caused by other microorganisms. Signs of sepsis were described at presentation in 35 patients (59% of those with available information). Transthoracic echocardiography (TTE) was sufficient for a diagnosis in 54 patients (68.3%), with transesophageal echocardiography (TEE) being essential for diagnosis in 25 cases (31.7%). Vegetations were large, with a median maximum size of 19.5 mm (IQR: 14–30 mm). Pulmonary septic emboli were present in 49 patients (93% of those with available information).

### 3.4. Treatment and Outcomes

Table 2 lists the treatments provided and clinical results. Forty-three (54.4%) patients underwent cardiac surgery, including 36 for pulmonary valve replacement and six for valve repair (one case with missing information), and four (5%) underwent percutaneous procedures, specifically, the removal of vegetations using on-circuit mechanical aspiration devices. Six (7.6%) patients died during hospitalization. Follow-up information was available for only 19 cases (26% of survivors), with a median follow-up of 12 months (IQR: 3–12 months).

### 3.5. Global Description of Fatal Cases

Of the six deceased patients, three had CHD and three belonged to the No-CHD/IDU group. The infections were caused by MRSA (two cases), *Streptococcus pneumoniae*, *Streptococcus anginosus*, *Klebsiella pneumoniae*, and blood-culture-negative IE in one case each. Pulmonary emboli were identified in the four cases for which information was available. In addition to medical treatment, one patient underwent PMA and two valvular surgeries (bioprosthetic replacements). In one case, the family declined surgery due to a poor prognosis, whereas in the remaining two cases, there is no detailed explanation as to why it was not evaluated and/or carried out.

### 3.6. Comparison of Cases According to Risk Factor

There were important differences based on evaluations of predisposing risk factors (see Table 3). Specifically, a higher median age in patients of the No-CHD/IDU group, greater gender parity in the patients with CHD, more staphylococcal etiology in IDU patients, and a trend towards more enterococcal infections in the No-CHD/IDU group were observed. Although numerically, the proportion of IDU patients received less cardiac surgery, there were no statistical differences between the groups in either the approach or hospital mortality. It is noteworthy, however, that no IDU patient died.

## 4. Discussion

The current systematic review includes 79 cases of adult patients with isolated PV-IE reported in the past 5 years. We found that patients were predominantly middle-aged men, with congenital heart disease (CHD) and IDU as risk factors, while almost 42% of cases did not have either of them (No-CHD/IDU group). Relevant differences across risk factor subgroups were observed, with a higher median age in patients of the No-CHD/IDU group, greater gender parity in patients with CHD, and more frequent staphylococcal etiology in patients with IDU. From the diagnosis standpoint, up to one-third of cases required TEE for a definite diagnosis while pulmonary and septic emboli were highly prevalent. Finally, more than half of patients underwent surgical intervention with an overall favorable short-term prognosis.

As described in previous publications, isolated pulmonary IE is uncommon, with percentages ranging from less than 1% [2] to 2.4% [1,3], and isolated involvement probably being more unique. Possible reasons include the low-pressure gradient within the right heart with less endothelial stress; less endothelial damage caused by intravenous drugs in relation to the tricuspid valve [82]; lower prevalence of right-sided congenital malformations; lower oxygen content in venous blood, which is less supportive of bacterial growth; and differences in the vascularization of the right heart endothelium [83]. Nevertheless, our review found 79 cases between 2020 and 2025, and so, even though it is a rare entity, clinicians should maintain a high index of suspicion in the presence of a clinical profile of right-sided IE.

Since we excluded pediatric patients and infections associated with previous cardiac surgeries, prosthetic materials, CIED, and long-term venous catheters from our systematic review, the cases we included do not reflect the overall epidemiological picture of right-sided IE. Nevertheless, it was clear that a significant proportion of cases had underlying CHD, with IE often being the first clinical manifestation for adult patients unaware of this predisposing condition. Another large group consisted of patients with IDU, a population that often classically presents with right-sided IE [82,84] and may have solitary involvement of this valve, which should be suspected when there is *S. aureus* bacteremia of an unclear source, pulmonary embolism, and a non-diagnostic echocardiogram.

Finally, we found a sizable percentage of patients without classic risk factors, as previously described [15,83,85]. This group, which could be considered a subgroup within a category that some authors coined as “3 noes RSIE” (no left-sided, no drug users, no CIED) [86], has peculiar clinical characteristics: they are generally male, middle-aged, community-acquired infections, and their etiologies may differ from those observed in the other groups. In our experience, *E. faecalis* tended to be more frequent in this group, while *S. aureus* was detected significantly more often in the IDU population. The finding that the median age of patients in the non-CHD/IDU group was 20 years more than that of the other two groups is remarkable. The association of *E. faecalis* with IE in older adults is well documented and explains, in part, the sustained increase of this microorganism in developed countries in recent years, currently ranking third in frequency among pathogens causing IE [87,88].

Probably due to selective reporting bias, the cases included in our review showed large vegetations, which could explain the apparently good performance of TTE, which proved sufficient for diagnosis in two-thirds of cases. However, this could also reflect advantages of the transthoracic approach due to certain anatomical peculiarities of the pulmonic valve [2,83]. Despite this, TEE was indispensable for definite diagnosis in up to one-third of patients, highlighting that both techniques are complementary. Furthermore, it is noteworthy that pulmonary emboli were common. In this regard, the possibility of pulmonary emboli should be considered in patients with apparent pneumonia and concomitant bacteremia caused by unusual respiratory pathogens (viridans group streptococci, *E. faecalis*, or *S. aureus*), bearing in mind that a TTE alone may not adequately assess the pulmonic valve.

Although RS-IE is generally considered a condition of primarily medical management, more than half of the isolated PV-IE cases included herein required cardiac surgery, primarily for valve replacement, likely due to selection bias for publication purposes. In the largest study published to date evaluating surgical interventions in PV-IE [89], the clinical outcomes were remarkably good (in-hospital mortality of 5.5%), with no significant differences between replacement vs. repair. In the cases included in our review, overall mortality was low (7.6%), similar to that described in previous observations [2,8], with no deaths among patients with IDU. While this latter finding may be attributed to the younger age and lower comorbidity of PWID patients [3,84], underreporting due to publication bias or loss to follow-up in this group cannot be completely excluded.

This systematic review has several limitations. First, as this is a systematic review primarily of case reports, there is an inherent risk of bias due to selective reporting. Furthermore, the included manuscripts present a heterogeneous description of the interventions and significant shortcomings in the follow-up data after hospital discharge, which is commonplace in retrospective investigations. Despite these limitations, this work offers a comprehensive review of published cases of this rare syndrome.

## 5. Conclusions

In summary, our systematic review detected differences among risk factor subgroups, with a higher median age in the non-CHD/IDU group, greater gender parity in CHD patients, and a higher prevalence of staphylococcal etiology in patients with IDU, although treatment approaches and mortality did not differ significantly. This review suggests an overall favorable prognosis for this disease, although potential reporting bias warrants caution and underscores the need for larger, systematically collected datasets to better characterize outcomes.

## Figures and Tables

**Table 1:** Demographics, risk factors, etiology, clinical presentation, and imaging.

Study_ID	Design	N	Age	Sex	CHD	IDU	Etiology	Sepsis	TTE	TEE	Lung Involvement—Description
Zhang 2021 [12]	CR	1	28	Male	No	No	MSSA	Yes	Two masses (14 × 13 mm and 11 × 16 mm), very mobile and attached to the PV		Multiple patchy and large lesions with cavities in both lungs
Zhang 2023 [13]	CR	1	43	Female	PDA	No	Negative BC	No	Vegetation (8 × 8 mm) on the PV		Not available
Xiong 2025 [14]	CR	1	27	Male	No	Yes	MSSA	Yes	A large PV vegetation (32 mm). There was no PV regurgitation and the right ventricular systolic function was normal		Septic embolism within the main PA as well as multiple bilateral upper-lobe peripheral soft tissue densities likely representing focal septic emboli
Whitehead 2023 [15]	CR	1	Early 70s	Male	No	No	*E. faecalis*	No	Large vegetation on his native PV with severe PR	Confirmatory	Chest CT during admission showed a 3 cm cavitating mass
Velez 2025 [16]	CR	1	22	Female	VSD	No	*S. gordonii*	No	No obvious vegetations	Revealed a 4 mm vegetation attached to the arterial side of the PV with trace PR. All other heart valves were unaffected	Not available
Vâta 2025 [17]	CR	1	69	Male	No	No	*E. faecalis*	Yes	Two PV vegetations: 21 mm on the posterior cusp and 17 mm on the anterior cusp. Moderate-to-severe PR	Confirmatory	No
Valsky 2024 [18]	CR	1	49	Male	No	No	*S. agalactiae*, *S. haemolyticus* and *S. pettenkoferi*	No	Isolated PV vegetation of 40 mm	Confirmatory	Multiple peripheral pulmonary emboli
Tominaga 2022 [19]	CR	1	28	Male	CCTGA, VSD, and PS	No	*A. defectiva*	Yes	Mobile vegetation of 26 mm at the PV		Multiple septic pulmonary emboli
Toader 2020 [20]	CR	1	36	Male	PDA with bidirectional shunt	No	Negative BC	Yes	Large vegetations located on PV and PR and dilation of the PV. Fistula between RVOT and aorta	Confirmatory	Pulmonary embolism
Stefaniak 2024 [21]	CR	1	45	Male	No	No	*H. parainfluenzae*	Yes	Mass of 6 × 10 mm in the RVOT with a connection to the PV. Moderate PR	Mass increased to 8 × 12 mm	Not available
Srdanovic 2023 [22]	CR	1	36	Female	Congenital PA stenosis	No	*Staphylococcus* spp. and *Corynebacterium* spp.	No	Multiple floating vegetations at the RVOT, PV, and the walls of the PA		Multiple septic pulmonary emboli
Smits 2020 [23]	CR	1	68	Male	Small ASD type 2 with left-to-right shunt as well as a moderate PS	No	*E. faecalis*	Not available	Mobile structure on the PV with an increased transpulmonary valve gradient	Confirmatory. Severe PR	Not available
Shah 2021 [24]	CR	1	57	Male	No	Yes	*S. pneumoniae*	Yes	PV vegetation and severe global hypokinesis. No vegetation was noted on other valves		Not available
Rao 2022 [25]	CR	1	50	Male	Noonan syndrome with dysplastic PV and severe PS	No	*S. haemolyticus*	Yes	Mobile vegetation on the PV with moderate PR. Pulmonary abscess of 3 mm extending into the RVOT		On chest X-ray, there was a patch in the left lower lobe
RajaShariff 2020 [26]	CR	1	23	Male	0.8 cm perimembranous, restrictive VSD	No	MSSA	Not available	No visible vegetations or masses were demonstrated	Abnormal PV morphology, with evidence of anterior cusp prolapse. Multiple hyperechoic structures in the entirety of the anterior cusp, with the largest measuring 5 × 6 mm in size. Severe PR	Not available
Platz 2020 [27]	CR	1	39	Male	No	Yes	*S. dysgalactiae*	Yes	No signs of endocarditis	A round and mobile vegetation of 14 × 14 mm in size on PV, mid- to high-grade PR	Bilateral diffuse pulmonary infiltrates
Placido 2020 [28]	CR	1	45	Female	Morphologic LV-type univentricular heart with both atrioventricular valve openings. The great arteries were transposed, with the PA arising from the morphologic LV and the aorta arising from a rudimentary chamber (RV). The LV ejected into the outflow tract through an interventricular septum defect	No	*K. pneumoniae*	Not available	A large mobile vegetation was seen in the PV		Chest CT showed septic pulmonary emboli
Paudel 2025 [29]	CR	1	68	Male	No	No	*B. quintana* (serology)	No	Thickening of the PV	19 mm vegetation on PV and moderate PR	Not available
Patrassi 2022 [30]	CR	1	64	Male	No	No	*S. gallolyticus*	No	Lone PV endocarditis with large mobile vegetations swinging between RVOT and PA		Massive pulmonary embolism
Patel 2024 [31]	CR	1	UK	Not available	No	No	*E. faecalis*	Yes	PV mass, right ventricular dilation, and elevated PA systolic pressure	Confirmed a 24 × 14 mm PV vegetation, severe PR, and severe RA enlargement leading to acute right heart failure	Repeat chest CT revealed septic pulmonary emboli
Patel 2024 [32]	CR	1	32	Female	No	Yes	Negative BC	Yes		PV vegetations	Multiple cavitary lesions throughout the lungs and dense consolidation in the right midlung field
Patel 2022 [33]	CR	1	37	Male	No	Yes	MSSA	No	Mobile density on PV measuring 18 × 8 mm	Persistent vegetation with exponential growth (50 mm)	Numerous pulmonary cavitary lesions
Parekh 2025 [34]	CR	1	31	Male	No	No	*S. haemolyticus*	No	9 mm PV vegetation	18 mm vegetation on the PV associated with a flail leaflet, with an otherwise preserved ejection fraction. PFO	Right heart strain, ground-glass opacifications in the right lower lobe of the lung
Parato 2022 [35]	CR	1	33	Male	No	Yes	MSSA	No	Two giant, elongated, and highly mobile PV vegetations, with the largest one measuring 50 × 10 mm, with torrential PR	Excluded additional complications or other valve involvement	Chest X-ray demonstrated multiple-site basal pneumonia and Chest CT revealed multiple septic emboli as cavitary and precavitary lesions
NourElHouda 2025 [36]	CR	1	43	Female	Hemodynamically significant PDA	No	*S. sanguinis* and *S. mitis*	No	A mobile, hyperechogenic vegetation measuring 35 mm was visualized on the PV, located on the PA side. No significant PR		Pulmonary septic emboli
Nguyen 2021 [37]	CR	1	85	Female	No	No	MSSA	Not available	Negative for valvular vegetations	Revealed 7 × 9 mm oscillating mass at the left cusp of the PV	Not available
Navarrete 2020 [38]	CR	1	35	Female	No	Yes	MRSA	Yes	Extensive mobile 37 × 5 mm vegetation on the PV with moderate to severe PR		Multiple small pulmonary emboli
Nahhal 2023 [39]	CR	1	81	Male	No	No	*S. oralis*	Not available	PR, which was considered more severe compared with an old TTE. PV vegetation measuring 14 × 9 mm		Chest CT showed bilateral scattered lung nodules
Munawar 2024 [40]	CR	1	37	Male	Small perimembranous VSD with a left-to-right shunt	No	Negative BC	Yes	Vegetations in the RVOT and on the PV		Multifocal areas of consolidation in the lungs, particularly on the left side
Monk 2023 [41]	CR	1	27	Male	No	Yes	MSSA	Yes	PV regurgitation, with high suspicion for valvular vegetation	PV endocarditis, with a 14 × 10 mm vegetation on the PV, and moderate PR	Embolus in the right lower lobe PA with septic bilateral pulmonary emboli
Ricci 2024 [42]	CR	1	76	Male	No	No	*S. gallolyticus*	Not available	Large vegetation on the PV (13 × 9 mm) with moderate PR		Not available
Lopez-Mora 2025 [43]	CR	1	34	Male	Restrictive perimembranous VSD, with a left-to-right shunt	No	MRSA	Yes		Two vegetations were visualized on the PV, measuring 20 × 8 mm and 17 × 9 mm. Trivial to mild PR	Multiple peribronchovascular consolidations. A focal consolidation with a reverse halo sign was noted in the posterior basal segment of the right lower lobe. Additionally, multiple randomly distributed bilateral pulmonary nodules
Lim 2022 [44]	CR	1	40	Male	Perimembranous VSD	No	*S. gordonii*	No	Highly mobile mass at the PV measuring 23 × 12 mm with free-flow PR		Embolization involving bilateral posterobasal segments of the lungs, which caused septic atelectasis
Kulahcioglu 2022 [7]	CR	1	23	Male	No	No	MRSA	Yes	Flail PV with 37 and 28 mm mobile multiple vegetations. Severe acute PR		Multiple pneumonic infiltrates were observed in the chest X-ray
Kisling 2024 [45]	CR	1	52	Male	Congenital supravalvular PS	No	*S. agalactiae*	Yes	PV regurgitation and moderate supravalvular PS without evidence of IE	PV thickening, a 21 × 7 mm vegetation, and prolapse of the left and anterior PV cusps causing severe PR	No septic emboli
Khosravi 2020 [46]	CR	1	47	Male	Severe PS	No	*Streptococcus* spp.	No	No valve vegetations	Large mobile mass (15 × 18 mm) on arterial side of PV and another very large mobile mass (25 × 9 mm) was attached to the luminal of main PA	Large cavitary lesion at right upper lobe in favor of septic emboli
Alan 2020 [47]	CR	1	65	Male	No	No	*A. urinae*	No		3 cm PV vegetation. Absence of significant PR.	Pulmonary emboli
Iturriagagoitia 2024 [48]	CR	1	64	Male	No	No	*E. faecalis*	Yes	Large and mobile masses attached to the PV (9 × 19 mm) prolapsing into the RVOT. Massive PR	Confirmed these finding	Chest CT showed a large consolidation with air bronchogram in the right upper lobe as well as bilateral patchy subpleural consolidations in both lower lobes and bilateral pleural fluid. Pulmonary emboli not present
Ignatius 2023 [49]	CR	1	51	Male	Wide ostium secundum ASD measuring 17 mm with a large left-to-right shunt	No	MSSA	Yes	Large vegetation measuring 27 × 9 mm on the PV with trivial PR		Chest X-ray was significant for bilateral patchy homogenous opacities with bilateral pleural effusion. Angio CT with filling defects in the segmental branch of the lobar branches of right pulmonary artery and peripheral multifocal scattered areas of consolidation with surrounding ground glass opacities suggestive of pulmonary infarcts from showering of emboli
Huynh 2025 [50]	CR	1	31	Male	No	No	*S. haemolyticus*	Yes	A 9 mm echogenic mass on the PV, PR, and dilated RV	A 18 mm vegetation with flail pulmonic leaflets and severe PR without abnormalities of the other valves	Right lower lobe segmental pulmonary embolism, pulmonary infarction
Hussein 2024 [51]	CR	1	30	Female	Restrictive perimembranous VSD	No	*K. pneumoniae*	Yes	Perimembranous VSD measuring 7 mm with left to right shunt. The PV showed 2 large highly mobile masses (28 × 12 mm and 24 × 9 mm). Severe PR		Bilateral consolidation and cavitations suggestive of septic pulmonary emboli
Hemli 2020 [52]	CR	1	27	Male	No	Yes	MSSA	Not available	Large mobile mass on the PV associated with severe PR. Pulmonic root abscess		Multiple septic pulmonary emboli
Haydon 2024 [53]	CR	1	51	Male	No	No	MSSA	Yes	Unremarkable	Multiple PV vegetations (largest 8 mm) and moderate PR	Chest CT with contrast displayed an acute right lower lobe pulmonary embolism and multiple scattered cavitary lesions, with the largest measuring 60 mm in the left upper lobe
Hajsadeghi 2024 [54]	CR	1	42	Male	CCTGA with PV stenosis	No	*Brucella spp* (serology)	No	A 19 × 17 mm mobile mass on the atrial side of the PV, which was connected to the root of the main PA		Not available
Goldstein 2023 [55]	CR	1	53	Male	No	No	MRSA	Yes	Vegetation on PV measuring 10 × 3 mm	Vegetation on PV measuring 21 × 12 mm with severe PR	Not available
Gizaw 2024 [56]	CR	1	52	Male	No	No	Negative BC	Yes	There was 10 × 12 mm sized vegetation on PV. Thickened and scalloped PV suggestive of PS, and moderate PR		Not available
Ghanshani 2020 [57]	CR	1	28	Male	No	Yes	*S. viridans*	Yes	A 18 × 12 mm vegetation on the PV complicated by leaflet destruction and severe PR		Chest CT demonstrated acute right heart strain, bilateral pulmonary emboli, and regions of lung infarct
Garatti 2023 [58]	CR	1	50	Male	No	Yes	MSSA	Not available	Huge mobile mass (24 × 17 mm) adherent to the PV		Chest CT scan showed bilateral interstitial pneumonia with ground-glass opacities, and superimposed right lower lobe pneumonia, probably related to septic embolization
Galuszka 2023 [59]	CR	1	74	Male	No	No	MSSA	Yes	Signs of right ventricular overload. No signs of endocarditis were detected	Masses on all cusps of the PV. A large (19 × 12 mm), mobile, ribbon-shaped vegetation was protruding into the RVOT	Multiple small consolidations, suggesting septic emboli
Funabashi 2023 [60]	CR	1	58	Male	No	No	MSSA	Yes	Small verrucous finding on the right ventricular side of the PV	The finding was not clear on TEE. An electrocardiography-gated cardiac CT showed the verrucae on the PV	No
FernandezValledor 2020 [61]	CR	1	35	Male	No	Yes	MSSA	Yes	A big vegetation (40 × 10 mm) on the PV that caused moderate PR		Chest X-ray showed a necrotizing bilateral pneumonia that was confirmed with the presence of cavitated images in the Chest CT
Fernandes 2025 [62]	CR	1	59	Male	No	No	MSSA	Yes	No evidence of valvular vegetation	Isolated PV vegetation measuring 20 × 18 mm causing severe PR and RV dilation	Bilateral areas of consolidation and cavitation in the right upper lobe
Felix 2024 [63]	CR	1	31	Female	No	Yes	MSSA	Not available	Isolated large irregularly shaped (30 × 15 mm) mass on the PV		Chest CT showed moderate-to-large embolic load bilaterally with scattered peripheral nodular densities and consolidations
Eugenio 2024 [64]	CR	1	85	Male	No	No	*E. faecalis*	No	A large mass on the PV inducing mild stenosis and PR		Bilateral multifocal pneumonia due to septic embolization
Doyle 2024 [65]	CR	1	66	Male	No	Yes	MRSA	Yes	Inconclusive	17 × 8 mm echodense mass attached to the PV’s anterior leaflet	Not available
Darwish 2025 [66]	CR	1	43	Female	Secundum ASD	No	*A. fumigatus*	No	40 × 16 mm multi-lobulated, multi-cystic mass on the PV, causing RVO obstruction		Chest CT demonstrated multiple bilateral pulmonary nodules
Ciampi 2024 [67]	CR	1	66	Male	No	No	Negative BC	no	Two iso-anechoic masses adherent to the PV, predominantly on the anterior and left cusps		Right segmental pulmonary embolism and associated pulmonary infarction
Chung 2024 [68]	CR	1	46	Male	No	Yes	Not available	Not available		Large vegetation on the PV with severe PR	Not available
Casey 2022 [69]	CR	1	73	Male	No	No	Negative BC (*S. constellatus* in pleural fluid)	Yes	PV vegetation measuring 13 × 9 mm, a small pericardial effusion, and mild PR without any involvement of other heart valves		Large left thoracic fluid collection (20 × 13 × 10 cm) as well as a large abnormality, which was concerning for a pulmonary embolism arising from the PV. Septic pulmonary microembolization
Biesboer 2021 [70]	CR	1	Elderly	Not available	No	No	*E. faecalis*	No	A large vegetation on the right PV leaflet	Confirmatory	No emboli
Berrajaa 2025 [71]	CR	1	33	Male	Perimembranous VSD	No	*S. constellatus*	Yes	Left-to-right shunt (a gradient of 52 mmHg), as well as a PV bearing multiple vegetations, the largest measuring 12 × 7 mm, leading to severe PR	Confirmatory	Mycotic aneurysm of the superior segmental artery of the right upper lobe
Beam 2021 [72]	CR	1	34	Male	VSD	No	Negative BC	Not available	A small mobile echodensity just inferior to the PV annulus with mild right ventricle dilation and mildly reduced systolic function		Multifocal, subsegmental pulmonary emboli without right heart enlargement
Barrios 2024 [73]	CR	1	29	Male	No	No	*F. solani*	No		New PV vegetations	Not available
Appiah-Kubi 2024 [74]	CR	1	47	Male	No	No	MRSA	No	Reduced ejection fraction but no valvular issues	A large (36 × 14 mm), mobile echodensity at the PV’s ventricular side	Not available
Antoun 2020 [75]	CR	1	56	Male	No	No	MRSA	No		Large mass compatible with vegetation was seen over the PV measured ~19 × 9 mm	Patchy lung infiltration in both sides compatible with pneumonia. Increase in size of the existing nodules and cavitations, as well as multiple new nodules
Ang 2025 [76]	CR	1	39	Male	No	No	MSSA	No	No evidence of PV endocarditis	Mobile vegetation on the ventricular aspect of the PV without PR	Areas of internal cavitation, suspicious of septic emboli
Ali 2020 [77]	CR	1	Young	Female	Perimembranous VSD	No	*K. kristinae*	No	A small 7 mm vegetation attached to PV	Confirmed findings	Not available
Al-Kourainy 2020 [78]	CR	1	50	Male	No	No	*S. pneumoniae*	Yes	A large, highly mobile vegetation on the PV with moderate PR, RV hypokinesis, and dilatation		Chest CT showed septic pulmonary emboli
Akkawi 2023 [79]	CR	1	40	Male	No	Yes	MSSA	Yes	A 32 mm PV vegetation with severe PR	Initially inconclusive	Multifocal pneumonia
Mohamed 2022 [80]	CS	1 *	65	Male	No	No	MSSA	Yes	Severe PR secondary to large vegetations and a dilated, severely impaired RV		Several small lung abscesses consistent with a diagnosis of septic pulmonary emboli
Sharma 2021 [5]	CS	4 **	18	Male	VSD and PS	No	Negative BC	Not available	Mobile mass on PV, protruding in PA (flail PV), PR		Not available
Sharma 2021 [5]	CS		22	Male	Tetralogy of Fallot	No	Negative BC	Not available	Mobile mass on PV, mild PR		Not available
Hicklin 2020 [6]	CS	7 ***	32	Male	No	Yes	MSSA—*A. odontolyticus*	Not available	Positive findings (not described)	Not performed	Not available
Hicklin 2020 [6]	CS		36	Male	No	Yes	MRSA—*K. pneumoniae*	Not available	No findings	Positive findings (not described)	Not available
Hicklin 2020 [6]	CS		27	Female	No	Yes	MRSA	Not available	No findings	Positive findings (not described)	Not available
Hicklin 2020 [6]	CS		46	Female	No	Yes	MRSA	Not available	No findings	Positive findings (not described)	Not available
Hicklin 2020 [6]	CS		44	Male	No	Yes	Negative BC	Not available	No findings	Positive findings (not described)	Not available
Hicklin 2020 [6]	CS		24	Female	No	Yes	MRSA	Not available	No findings	Positive findings (not described)	Not available
Hicklin 2020 [6]	CS		57	Male	No	No	*S. anginosus*	Not available	Positive findings (not described)	Positive findings (not described)	Not available

Notes: * One case excluded (mitral involvement); ** eight cases excluded (no PFO); *** cardiovascular risk factors: Diabetes, Hypertension, Smoking. Acronyms: ASD: Atrial septal defect; BC: blood culture; CCTGA: congenitally corrected transposition of the great arteries; CHD: congenital heart disease; CR: case report; CS: case series; CT: computed tomography; IDU: injection drug use; IE: infective endocarditis; LV: left ventricle; PA: pulmonary artery; PDA: patent ductus arteriosus; PFO: patent foramen ovale; PS: pulmonary stenosis; PR: pulmonary regurgitation; PV: pulmonary valve; RV: right ventricle; RVOT: Right ventricular outflow tract; TEE: transesophageal echocardiography; TTE: transthoracic echocardiography; VSD: ventricular septal defect. A. odontolyticus: *Actinomyces odontolyticus*; B. quintana: *Bartonella quintana*; F. solani: *Fusarium solanii*; H. parainfluenzae: *Haemophylus parainfluenzae*; K. kristinae: *Kokuria kristinae*; K. pneumoniae: *Klebsiella pneumoniae*; MRSA: Methicillin-resistant *Staphylococcus aureus*; MSSA: Methicillin-susceptible *Staphylococcus aureus*; E. faecalis: *Enterococcus faecalis*; S. for haemolyticus/pettenkoferi: *Staphylococcus;* S. (for agalactiae/dysgalactiae/constellatus/gallolyticus/gordonii/mitis/pneumoniae/sanguinis): *Streptococcus*.

**Table 2:** Procedures, outcomes, and follow-up.

Study_ID	Procedure	Description	In-Hospital Mortality	Follow-Up	Maximum Follow-Up (Months)
Zhang 2021 [12]	Cardiac Surgery	PV replacement with Saint Jude bioprosthetic valve (23 mm), vegetation removal, and right pulmonary thromboendarterectomy	No	No	
Zhang 2023 [13]	No		No	Yes	12
Xiong 2025 [14]	No		No	No	
Whitehead 2023 [15]	Cardiac Surgery	Pulmonary homograft	No	No	
Velez 2025 [16]	No		No	No	
Vâta 2025 [17]	Cardiac Surgery	PV replacement using a Magna Ease (25 mm)	No	Yes	12
Valsky 2024 [18]	Cardiac Surgery	PV replacement with a cryopreserved homograft (29 mm)	No	No	
Tominaga 2022 [19]	Cardiac Surgery	PV replacement using a 19 mm Epic valve (Abbott, CA, USA)	No	Yes	12
Toader 2020 [20]	Cardiac Surgery	Debridement, vegetation excision with PV replacement, relief of RVOT, fistula closure with pericardial patch, and ligature of PDA	No	No	
Stefaniak 2024 [21]	PMA	Angiovac^TM^ procedure. Vegetation suction under the PV	No	Yes	6
Srdanovic 2023 [20]	Cardiac Surgery	Homograft implantation	No	Yes	60
Smits 2020 [23]	No		No	No	
Shah 2021 [22]	No		No	No	
Rao 2022 [25]	Cardiac Surgery	PV replacement with a 21 mm Hancock II porcine heart valve with excision of the aneurysmal anterior wall of the PA with pericardial patch plasty	No	No	
Raja Shariff 2020 [26]	Cardiac Surgery	VSD closure and PV replacement in another institution	No	No	
Platz 2020 [27]	No		No	No	
Placido 2020 [28]	Cardiac Surgery	Vegetation removal and PV replacement by a bioprosthesis	Yes	No	
Paudel 2025 [29]	Cardiac Surgery	Not described	No	No	
Patrassi 2022 [30]	Cardiac Surgery	A Magna Ease bioprosthetic valve was implanted	No	No	
Patel 2024 [31]	Cardiac Surgery	Urgent bioprosthetic PV replacement, pericardial patch augmentation, and coronary artery bypass grafting	No	No	
Patel 2024 [32]	No		No	No	
Patel 2022 [33]	Cardiac Surgery	Vegetectomy	No	No	
Parekh 2025 [34]	Cardiac Surgery	PV replacement using a bioprosthetic valve, pericardial patch augmentation of the PA, and primary closure of his PFO	No	No	
Parato 2022 [35]	Cardiac Surgery	Implantation of a biological valved conduit model NR-2000C (Shelhigh, Inc., Milburn, NJ, USA)	No	Yes	3
NourElHouda 2025 [36]	Cardiac Surgery	Closure of the PDA and replacement of the dilated ascending aorta (elective; 3 months later). No residual vegetations were found intraoperatively	No	Yes	3
Nguyen 2021 [37]	No		No	No	
Navarrete 2020 [38]	Cardiac Surgery	Bioprosthetic PV replacement and PA reconstruction with autologous pericardial patch	No	No	
Nahhal 2023 [39]	No		No	No	
Munawar 2024 [40]	No		No	Yes	1
Monk 2023 [41]	No		No	No	
Ricci 2024 [42]	No		No	No	
Lopez-Mora 2025 [43]	PMA	Aspiration of the vegetations with a large-caliber suction equipment (16 Fr Lightning Flash™ (Penumbra, Inc., Alameda, CA, USA) aspiration system)	Yes	No	
Lim 2022 [44]	Cardiac Surgery	Pericardial bioprosthetic PV replacement and VSD closure	No	Yes	12
Kulahcioglu 2022 [7]	Cardiac Surgery	Bioprosthesis PV replacement and surgical expansion of the main PA and RVOT by using a pericardial patch	Yes	No	
Kisling 2024 [45]	Cardiac Surgery	PV/RVOT pericardial patch repair with implantation of a 29 mm Edwards Magna Ease valve	No	Yes	Not available
Khosravi 2020 [46]	Cardiac Surgery	PV was replaced with a 23 mm On-X valve. RVOT repair	No	Yes	3
Alan 2020 [47]	Cardiac Surgery	Bioprosthetic PV replacement	No	No	
Iturriagagoitia 2024 [48]	Cardiac Surgery	After resection of all the cusps with preservation of the annulus, a PV replacement was performed using a bioprosthesis	No	No	
Ignatius 2023 [49]	Cardiac Surgery	ASD closure with PV vegetectomy and valve repair	No	Yes	6
Huynh 2025 [50]	Cardiac Surgery	PV replacement	No	No	
Hussein 2024 [51]	Cardiac Surgery	Complete excision of the PV and infected tissue at the RVOT. Reconstruction of the pulmonary root using a 27 mm Freestyle valved conduit	No	Yes	12
Hemli 2020 [52]	Cardiac Surgery	Aggressive debridement. An aortic homograft was used to reconstruct the RVOT, the PV, and the proximal main PA	No	No	
Haydon 2024 [53]	No		No	No	
Hajsadeghi 2024 [54]	No		No	No	
Goldstein 2023 [55]	Cardiac Surgery	Debridement of PV and RV. PV replacement	No	No	
Gizaw 2024 [56]	No		No	Yes	3
Ghanshani 2020 [57]	Cardiac Surgery	PV replacement	No	Yes	12
Garatti 2023 [58]	Cardiac Surgery	A 25 mm Edwards Perimount bioprosthesis (Edwards Lifesciences, Irvine, CA, USA) was implanted, enlarging the RVOT with a heterologous pericardial patch. Finally, a TV De-Vega annuloplasty was performed without a prosthetic ring	No	No	
Galuszka 2023 [59]	No		No	No	
Funabashi 2023 [60]	No		No	No	
FernandezValledor 2020 [61]	Cardiac Surgery	Vegetectomy; the posterior valve needed to be repaired	No	No	
Fernandes 2025 [62]	Cardiac Surgery	Bioprosthetic PV replacement	No	No	
Felix 2024 [63]	No		No	No	
Eugenio 2024 [64]	Cardiac Surgery	PV replacement with a biological prosthesis	No	No	
Doyle 2024 [65]	No		No	No	
Darwish 2025 [66]	Cardiac Surgery	PV along with vegetations in the RVOT were removed. Replacement with a bioprosthetic valve. Secundum ASD was also repaired	No	Yes	12
Ciampi 2024 [67]	Cardiac Surgery	Replacement of PV with a bioprosthesis and removal of the two neoformations	No	No	
Chung 2024 [68]	PMA	Transcatheter vacuum-assisted mass extraction using the AngioVac^TM^ system	No	No	
Casey 2022 [69]	No		No	No	
Biesboer 2021 [70]	Cardiac Surgery	PV excised and replaced with a 27 mm porcine bioprosthesis (elective; 3 years after the infection)	No	Yes	36
Berrajaa 2025 [71]	No		No	No	
Beam 2021 [72]	Cardiac Surgery	PV replacement and closure of the VSD	No	No	
Barrios 2024 [73]	No		No	Yes	7
Appiah-Kubi 2024 [74]	PMA	Attempts were futile due to the vegetation’s small size	No	No	
Antoun 2020 [75]	Cardiac Surgery	PV replacement using a LivaNova extra-large sutureless valve	No	No	
Ang 2025 [76]	Cardiac Surgery	PV vegetectomy	No	No	
Ali 2020 [77]	No		No	No	
Al-Kourainy 2020 [78]	No		Yes	No	
Akkawi 2023 [79]	Cardiac Surgery	PV replacement	No	No	
Mohamed 2022 [80]	Cardiac Surgery	Native PV was excised, and tissue thoroughly debrided. A bioprosthetic valve was implanted	No	No	
Sharma 2021 [5]	No		Yes	No	
Sharma 2021 [5]	Cardiac Surgery	VSD and RVOT patch repair	No	Yes	24
Hicklin 2020 [6]	No		No	No	
Hicklin 2020 [6]	No		No	No	
Hicklin 2020 [6]	No		No	No	
Hicklin 2020 [6]	No		No	No	
Hicklin 2020 [6]	No		No	No	
Hicklin 2020 [6]	No		No	No	
Hicklin 2020 [6]	No		Yes	No	

Acronyms: ASD: Atrial septal defect; PA: pulmonary artery; PDA: patent ductus arteriosus; PFO: patent foramen ovale; PMA: percutaneous mechanical aspiration; PV: pulmonary valve; RVOT: right ventricular outflow tract; TV: tricuspid valve; USA: United States of America; VSD: ventricular septal defect.

**Table 3:** Comparison of clinical characteristics according to risk factor group.

	CHD N = 24	IDU N = 22	No-CHD/IDU N = 33
Age (years)/Median (IQR)	37 (30–45)	35 (28–45)	57.5 (47–68) ^a^
Male Sex	16 (66.7%)	16 (72.7%)	30 (90.9%) ^b^
Etiology			
MSSA	2 (8.3%)	10 (45.5%) ^c^	8 (24.2%)
MRSA	1 (4.2%)	6 (27.3%) ^d^	4 (12.1%)
Streptococci	5 (20.8%)	3 (13.6%)	6 (18.2%)
*E. faecalis*	1 (4.2%)	0	6 (18.2%) ^e^
Sepsis at presentation	9 (37.5%)	10 (45.5%)	16 (48.5%)
Lung involvement	15 (62.5%)	13 (59.1%)	21 (63.6%)
Procedures			
Cardiac surgery	15 (62.5%)	8 (36.4%)	20 (60.6%)
PMA	1 (4.2%)	1 (4.5%)	2 (6.1%)
In-hospital mortality	3 (12.5%)	0	3 (9.1%)

^a^ *p* < 0.001 for No-CHD/IDU vs. CHD and for No-CHD/IDU vs. IDU. ^b^ *p* = 0.039 for No-CHD/IDU vs. CHD. ^c^ *p* = 0.007 for IDU vs. CHD. ^d^ *p* = 0.043 for IDU vs. CHD. ^e^ *p* = 0.071 for No-CHD/IDU vs. IDU. Acronyms: CHD: Congenital heart disease; IDU: injection drug use; IQR: interquartile range; No-CHD/IDU: patients without CHD and non-intravenous drug users; PMA: percutaneous mechanical aspiration; MRSA: methicillin-resistant *Staphylococcus aureus*; MSSA: methicillin-susceptible *Staphylococcus aureus*; *E. faecalis*: *Enterococcus faecalis*.

## Data Availability

The data underlying this article will be shared on reasonable request to the corresponding author.

## Supplementary Materials

The following supporting information can be downloaded at: https://www.mdpi.com/article/10.3390/microorganisms14061208/s1, File S1: Search strategies (according to the PRISMA-S extension); File S2: PRISMA 2020 Checklist.; File S3: Quality assessment of included articles; Table S1: Quality assessment of manuscripts (according to the Joanna Briggs Institute Critical Appraisal Checklist for case Reports).
