# Supplementary material for: Clinical Features of Adult Patients with Isolated Pulmonary Valve Endocarditis: A Systematic Review
Source: Microorganisms. 2026 May 27;14(6):1208. doi: 10.3390/microorganisms14061208 (PMC13303898; doi:10.3390/microorganisms14061208)
Supplement: Supplementary file 1 [file microorganisms-14-01208-s001.zip › microorganisms-4283514-supplementary.pdf]

Supplementary Material

File S1: Search strategies (according to the PRISMA-S extension)

Databases & Registers	# of initial hits
Central	2
Embase	1121
Medline	456
Scopus	933
Web of Science	446
Totals	2958

Duplicates initially removed by Covidence – 1034

Cochrane Central Register of Controlled Trials (CCTR) via Ovid (1991+)):

#	Query	Results from 4 Nov 2025
1	(endocarditis or infectiveendocarditis or endo-carditis).ab,hw,ti.	855
2	((pulmon* adj1 (valv* or cusp* or leaflet* or annulus)) or eustachian or chiari or (mural adj2 endocarditis)).ab,hw,ti. or (pulmon* adj2 IE).ti.	746
3	(non-tricuspid or nontricuspid or non-TVE or non-TVIE).ab,hw,ti.	1
4	or/2-3	747
5	endocardi*.ti,kw.	634
6	1 and 4 and 5	3
7	limit 6 to yr="2005 -Current"	2

Embase via Ovid (1974+):

#	Query	Results from 4 Nov 2025
1	exp endocarditis/	63,572
2	(endocarditis or infectiveendocarditis or endo-carditis).ab,kf,ti.	57,034
3	or/1-2	73,507
4	exp pulmonary valve/ or heart right atrium/ or exp heart right ventricle/ or ((pulmon* adj1 (valv* or cusp* or leaflet* or annulus)) or eustachian or chiari or (mural adj2 endocarditis)).ab,kf,ti. or (pulmon* adj2 IE).ti.	102,969
5	(non-tricuspid or nontricuspid or non-TVE or non-TVIE).ab,kf,ti.	24
6	or/4-5	102,991
7	3 and 6	3,632
8	((exp animal/ or animal experiment/ or nonhuman/) not (exp human/ or human experiment/)) or (exp juvenile/ not exp adult/)	10,558,443
9	7 not 8	3,165
10	endocardi*.ti,kf.	39,391
11	9 and 10	1,543
12	heart valve prosthesis/ or prosthe*.ti,kf.	99,033
13	11 not 12	1,369
14	limit 13 to yr="2005 -Current"	1,121

MEDLINE via Ovid (1946+ and Epub Ahead of Print, In-Process & Other Non-Indexed Citations and Ovid MEDLINE(R) Daily):

#	Query	Results from 4 Nov 2025
1	exp Endocarditis/	32,032
2	(endocarditis or infectiveendocarditis or endo-carditis).ab,kf,ti.	44,041
3	or/1-2	49,239
4	Pulmonary Valve/ or ((pulmon* adj1 (valv* or cusp* or leaflet* or annulus)) or eustachian or chiari or (mural adj2 endocarditis)).ab,kf,ti. or (pulmon* adj2 IE).ti.	27,681
5	(non-tricuspid or nontricuspid or non-TVE or non-TVIE).ab,kf,ti.	14
6	or/4-5	27,694
7	3 and 6	1,380
8	exp animals/ not humans/	5,390,789
9	7 not 8	1,335
10	endocardi*.ti,kf.	34,252
11	9 and 10	844
12	Heart Valve Prosthesis/ or prosthe*.ti,kf.	105,964
13	11 not 12	699
14	limit 13 to yr="2005 -Current"	456

Scopus via Elsevier (1788+):

(((TITLE-ABS-KEY ((endocarditis OR infectiveendocarditis OR endo-carditis))) AND ((TITLE-ABS-KEY ((pulmon* W/1 (valv* OR cusp* OR leaflet* OR annulus)) OR eustachian OR chiari OR (mural W/2 endocarditis)) OR TITLE (pulmon* W/2 IE)) OR (TITLE-ABS-KEY ((non-tricuspid OR nontricuspid OR non-TVE OR non-TVIE))))) AND NOT ((TITLE (prosthe* OR animal* OR veterinar* OR rat OR rats OR mouse OR mice OR murine OR cattle OR cow OR cows OR cattle OR bovine OR pig* OR porcine OR swine OR sheep OR ovine OR dog OR dogs OR canine* OR p*ediatric* OR child* OR neonat* OR newborn* OR infan* OR girl* OR boy* OR adolesc* OR teen* OR youth) OR KEY (prosthe* OR animal* OR veterinar* OR rat OR rats OR mouse OR mice OR murine OR cattle OR cow OR cows OR cattle OR bovine OR pig* OR porcine OR swine OR sheep OR ovine OR dog OR dogs OR canine* OR p*ediatric* OR child* OR neonat* OR newborn*

OR infan* OR girl* OR boy* OR adolesc* OR teen* OR youth) OR SRCTITLE (prosthe* OR animal* OR veterinar* OR rat OR rats OR mouse OR mice OR murine OR cattle OR cow OR cows OR cattle OR bovine OR pig* OR porcine OR swine OR sheep OR ovine OR dog OR dogs OR canine* OR p*ediatric* OR child* OR neonat* OR newborn* OR infan* OR girl* OR boy* OR adolesc* OR teen* OR youth)))) AND (TITLE (endocarditis) OR KEY (endocarditis)) AND Limit to 2005+

Web of Science Core Collection via Clarivate Analytics (Science Citation Index Expanded 1975+ & Emerging Sources Citation Index 2015+):

#9	#7 AND #8 AND Limit 2005+
#8	TI=(endocarditis) OR AK=(endocarditis)
#7	#5 NOT #6
#6	TI=(prosthe* or animal* or veterinar* or rat or rats or mouse or mice or murine or cattle or cow or cows or cattle or bovine or pig* or porcine or swine or sheep or ovine or dog or dogs or canine* or p$ediatric* or child* or neonat* or newborn* or infan* or girl* or boy* or adolesc* or teen* or youth) OR AK=(prosthe* or animal* or veterinar* or rat or rats or mouse or mice or murine or cattle or cow or cows or cattle or bovine or pig* or porcine or swine or sheep or ovine or dog or dogs or canine* or p$ediatric* or child* or neonat* or newborn* or infan* or girl* or boy* or adolesc* or teen* or youth) OR SO=(prosthe* or animal* or veterinar* or rat or rats or mouse or mice or murine or cattle or cow or cows or cattle or bovine or pig* or porcine or swine or sheep or ovine or dog or dogs or canine* or p$ediatric* or child* or neonat* or newborn* or infan* or girl* or boy* or adolesc* or teen* or youth)
#5	#1 AND #4
#4	#2 OR #3
#3	TS=(non-tricuspid or nontricuspid or non-TVE or non-TVIE)
#2	TS=((pulmon* NEAR/1 (valv* or cusp* or leaflet* or annulus)) or eustachian or chiari or (mural NEAR/2 endocarditis)) or TI=(pulmon* NEAR/2 IE)
#1	TS=(endocarditis or infectiveendocarditis or endo-carditis)

File S2: PRISMA 2020 Checklist

Section and Topic	Item #	Checklist item	Location where item is reported
**TITLE**			
Title	1	Identify the report as a systematic review.	Page 1
**ABSTRACT**			
Abstract	2	See the PRISMA 2020 for Abstracts checklist.	Page 1
**INTRODUCTION**			
Rationale	3	Describe the rationale for the review in the context of existing knowledge.	Page 2
Objectives	4	Provide an explicit statement of the objective(s) or question(s) the review addresses.	Page 2
**METHODS**			
Eligibility criteria	5	Specify the inclusion and exclusion criteria for the review and how studies were grouped for the syntheses.	Page 2
Information sources	6	Specify all databases, registers, websites, organisations, reference lists and other sources searched or consulted to identify studies. Specify the date when each source was last searched or consulted.	Page 2<br>Appendix 1 (Suppl. mat.)
Search strategy	7	Present the full search strategies for all databases, registers and websites, including any filters and limits used.	Appendix 1 (Suppl. mat.)
Selection process	8	Specify the methods used to decide whether a study met the inclusion criteria of the review, including how many reviewers screened each record and each report retrieved, whether they worked independently, and if applicable, details of automation tools used in the process.	Pages 2
Data collection process	9	Specify the methods used to collect data from reports, including how many reviewers collected data from each report, whether they worked independently, any processes for obtaining or confirming data from study investigators, and if applicable, details of automation tools used in the process.	Pages 2-3
Data items	10a	List and define all outcomes for which data were sought. Specify whether all results that were compatible with each outcome domain in each study were sought (e.g. for all measures, time points, analyses),	Pages 2-3

Section and Topic	Item #	Checklist item	Location where item is reported
		and if not, the methods used to decide which results to collect.	
	10b	List and define all other variables for which data were sought (e.g. participant and intervention characteristics, funding sources). Describe any assumptions made about any missing or unclear information.	Pages 2-3
Study risk of bias assessment	11	Specify the methods used to assess risk of bias in the included studies, including details of the tool(s) used, how many reviewers assessed each study and whether they worked independently, and if applicable, details of automation tools used in the process.	Page 7<br>Appendix 3 (Suppl. mat.)
Effect measures	12	Specify for each outcome the effect measure(s) (e.g. risk ratio, mean difference) used in the synthesis or presentation of results.	N/A
Synthesis methods	13a	Describe the processes used to decide which studies were eligible for each synthesis (e.g. tabulating the study intervention characteristics and comparing against the planned groups for each synthesis (item #5)).	Pages 2-3
	13b	Describe any methods required to prepare the data for presentation or synthesis, such as handling of missing summary statistics, or data conversions.	Pages 2-3
	13c	Describe any methods used to tabulate or visually display results of individual studies and syntheses.	Pages 2-3
	13d	Describe any methods used to synthesize results and provide a rationale for the choice(s). If meta-analysis was performed, describe the model(s), method(s) to identify the presence and extent of statistical heterogeneity, and software package(s) used.	Pages 2-3
	13e	Describe any methods used to explore possible causes of heterogeneity among study results (e.g. subgroup analysis, meta-regression).	Pages 2-3
	13f	Describe any sensitivity analyses conducted to assess robustness of the synthesized results.	Pages 2-3
Reporting bias assessment	14	Describe any methods used to assess risk of bias due to missing results in a synthesis (arising from reporting biases).	Pages 2-3
Certainty assessment	15	Describe any methods used to assess certainty (or confidence) in the body of evidence for an outcome.	Pages 2-3
**RESULTS**			
Study selection	16a	Describe the results of the search and selection process, from the number of records identified in the search to the number of studies	Page 3

Section and Topic	Item #	Checklist item	Location where item is reported
		included in the review, ideally using a flow diagram.	
	16b	Cite studies that might appear to meet the inclusion criteria, but which were excluded, and explain why they were excluded.	PRISMA chart (Figure 1)
Study characteristics	17	Cite each included study and present its characteristics.	Appendix 2 (Suppl. mat.)
Risk of bias in studies	18	Present assessments of risk of bias for each included study.	Appendix 3 (Suppl. mat.)
Results of individual studies	19	For all outcomes, present, for each study: (a) summary statistics for each group (where appropriate) and (b) an effect estimate and its precision (e.g. confidence/credible interval), ideally using structured tables or plots.	Appendix 2 (Suppl. mat.)
Results of syntheses	20a	For each synthesis, briefly summarise the characteristics and risk of bias among contributing studies.	Appendix 3 and Table S1 (Suppl. mat.)
	20b	Present results of all statistical syntheses conducted. If meta-analysis was done, present for each the summary estimate and its precision (e.g. confidence/credible interval) and measures of statistical heterogeneity. If comparing groups, describe the direction of the effect.	Page 3 and Tables 1, 2 and 3
	20c	Present results of all investigations of possible causes of heterogeneity among study results.	Appendix 3 and Table S1 (Suppl. mat.)
	20d	Present results of all sensitivity analyses conducted to assess the robustness of the synthesized results.	N/A
Reporting biases	21	Present assessments of risk of bias due to missing results (arising from reporting biases) for each synthesis assessed.	Appendix 3 and Table S1 (Suppl. mat.)
Certainty of evidence	22	Present assessments of certainty (or confidence) in the body of evidence for each outcome assessed.	Appendix 3 and Table S1 (Suppl. mat.)
**DISCUSSION**			
Discussion	23a	Provide a general interpretation of the results in the context of other evidence.	Page 19
	23b	Discuss any limitations of the evidence included in the review.	Page 19
	23c	Discuss any limitations of the review processes used.	Page 19
	23d	Discuss implications of the results for practice, policy, and future research.	Page 19
**OTHER INFORMATION**			

Section and Topic	Item #	Checklist item	Location where item is reported
Registration and protocol	24a	Provide registration information for the review, including register name and registration number, or state that the review was not registered.	N/A
	24b	Indicate where the review protocol can be accessed, or state that a protocol was not prepared.	N/A
	24c	Describe and explain any amendments to information provided at registration or in the protocol.	N/A
Support	25	Describe sources of financial or non-financial support for the review, and the role of the funders or sponsors in the review.	Page 20
Competing interests	26	Declare any competing interests of review authors.	Page 20
Availability of data, code and other materials	27	Report which of the following are publicly available and where they can be found: template data collection forms; data extracted from included studies; data used for all analyses; analytic code; any other materials used in the review.	Page 21

From: Page MJ, McKenzie JE, Bossuyt PM, Boutron I, Hoffmann TC, Mulrow CD, et al. The PRISMA 2020 statement: an updated guideline for reporting systematic reviews. BMJ 2021;372:n71. doi: 10.1136/bmj.n71

File S3: Quality assessment of included articles

The evaluation of the eight domains suggested by the Joanna Briggs Institute Critical Appraisal Checklist for Case Reports is detailed in Table 1 of the supplementary material. Since all but three of the included studies were case reports, 97% of the included studies presented a high risk of selective reporting bias. The basic clinical information can be considered robust: demographics (94%), medical history (85%), and current condition (81%) were adequately described. Diagnostic tests were detailed in 74% of the included studies. Interventions were well documented (97%), but post-intervention outcomes were the main limitation, being adequately reported in only 31%. Even so, 96% of the included studies offered takeaway lessons. Overall, the evidence obtained can be considered useful for generating hypotheses and guiding clinical decisions, but the limited follow-up data and the high risk of selective reporting bias require caution when interpreting the described clinical outcomes.

Table S1: Quality assessment of manuscripts (according to the Joanna Briggs Institute Critical Appraisal Checklist for case Reports)

Study ID	Risk of Selective reporting	Patient's demographic description	Patient's history description	Current clinical condition description	Diagnostic tests description	Intervention(s) description	Post-intervention description	Takeaway lessons
Zhang 2021	Yes	Yes	Yes	Yes	Yes	Yes	No	Yes
Zhang 2023	Yes	Yes	Yes	Yes	Unclear	Yes	Yes	Yes
Xiong 2025	Yes	Yes	Yes	Yes	Yes	Yes	No	Yes
Whitehead 2023	Yes	Unclear	Yes	Yes	Yes	Yes	No	Yes
Velez 2025	Yes	Yes	Yes	Yes	Unclear	Yes	No	Yes
Vâta 2025	Yes	Yes	Yes	Yes	Yes	Yes	Yes	Yes
Valsky 2024	Yes	Yes	Yes	Yes	Yes	Yes	No	Yes
Tominaga 2022	Yes	Yes	Yes	Yes	Yes	Yes	Yes	Yes
Toader 2020	Yes	Yes	Yes	Yes	Yes	Yes	No	Yes
Stefaniak 2024	Yes	Yes	Yes	Yes	Unclear	Yes	Yes	Yes
Srdanovic 2023	Yes	Yes	Yes	Yes	Yes	Yes	Yes	Yes
Smits 2020	Yes	Yes	No	No	Unclear	No	No	Unclear
Sharma 2021	No	Yes	Yes	Yes	Unclear	Yes	Yes	Yes
Shah 2021	Yes	Yes	Yes	Yes	Unclear	Yes	No	Yes
Rao 2022	Yes	Yes	Yes	Yes	Yes	Yes	No	Yes
RajaShariff 2020	Yes	Yes	Yes	Unclear	Unclear	Yes	No	Yes
Platz 2020	Yes	Yes	Yes	Yes	Yes	Yes	Yes	Yes
Placido 2020	Yes	Yes	Yes	Unclear	Yes	Yes	Unclear	Yes
Paudel 2025	Yes	Yes	Yes	Unclear	Unclear	Unclear	No	Yes
Patrassi 2022	Yes	Yes	Yes	Yes	Yes	Yes	No	Yes
Patel 2024	Yes	No	Yes	Yes	Yes	Yes	No	Yes
Patel 2024	Yes	Yes	Yes	Unclear	Yes	Yes	No	Yes
Patel 2022	Yes	Yes	Yes	Yes	Yes	Yes	No	Yes
Parekh 2025	Yes	Yes	Yes	Yes	Yes	Yes	No	Yes
Parato 2022	Yes	Yes	Yes	Yes	Yes	Yes	Yes	Yes
NourElHouda 2025	Yes	Yes	Yes	Yes	Yes	Yes	Yes	Yes
Nguyen 2021	Yes	Yes	Yes	Unclear	Unclear	Yes	No	Yes
Navarrete 2020	Yes	Yes	Yes	Yes	Yes	Yes	Yes	Yes
Nahhal 2023	Yes	Yes	Yes	Yes	Yes	Yes	No	Yes
Munawar 2024	Yes	Yes	Yes	Yes	Yes	Yes	Unclear	Yes
Monk 2023	Yes	Yes	Unclear	Yes	Yes	Yes	No	Yes
Mohamed 2022	Yes	Yes	Yes	Yes	Yes	Yes	No	Yes
Mirella 2024	Yes	Yes	Yes	Yes	No	Yes	No	Unclear
Lopez-Mora 2025	Yes	Yes	Yes	Yes	Yes	Yes	Yes	Yes
Lim 2022	Yes	Yes	Yes	Yes	Yes	Yes	Yes	Yes
Kulahcioglu 2022	Yes	Yes	Yes	Yes	Yes	Yes	Yes	Yes
Kisling 2024	Yes	Yes	Yes	Yes	Yes	Yes	Unclear	Yes
Khosravi 2020	Yes	Yes	Yes	Yes	Yes	Yes	Yes	Yes
John 2020	Yes	Yes	No	Unclear	Unclear	Yes	No	Yes
Iturriagagoitia 2024	Yes	Yes	Yes	Yes	Yes	Yes	No	Yes
Ignatius 2023	Yes	Yes	Yes	Yes	Yes	Yes	Yes	Yes
Huynh 2025	Yes	Yes	Yes	Yes	Yes	Yes	No	Yes
Hussein 2024	Yes	Yes	Yes	Yes	Yes	Yes	Yes	Yes
Hicklin 2020	No	Yes	No	No	Yes	Yes	Unclear	Yes
Hemli 2020	Yes	Yes	No	Unclear	Yes	Yes	No	Yes
Haydon 2024	Yes	Yes	Yes	Yes	Yes	Yes	No	Yes
Hajsadeghi 2024	Yes	Yes	Yes	Unclear	Unclear	Yes	No	Yes
Goldstein 2023	Yes	Yes	Yes	Yes	Unclear	Yes	No	Yes
Gizaw 2024	Yes	Yes	Yes	Yes	Unclear	Yes	Yes	Yes
Ghanshani 2020	Yes	Yes	Yes	Yes	Yes	Yes	Yes	Yes
Garatti 2023	Yes	Yes	Yes	Unclear	Yes	Yes	No	Yes
Galuszka 2023	Yes	Yes	Yes	Yes	Yes	Yes	No	Yes
Funabashi 2023	Yes	Yes	Unclear	Yes	Yes	Yes	No	Yes
FernandezValledor 2020	Yes	Yes	Yes	Yes	Yes	Yes	No	Yes
Fernandes 2025	Yes	Yes	Yes	Yes	Yes	Yes	No	Yes
Felix 2024	Yes	Yes	Yes	Unclear	Yes	Yes	No	Yes
Eugenio 2024	Yes	Yes	Yes	Yes	Yes	Yes	No	Yes
Doyle 2024	Yes	Yes	No	No	Unclear	Yes	No	Unclear
Darwish 2025	Yes	Yes	Yes	Yes	Yes	Yes	Yes	Yes
Ciampi 2024	Yes	Yes	Yes	Yes	Yes	Yes	No	Yes
Chung 2024	Yes	Yes	No	No	No	Yes	No	Yes
Casey 2022	Yes	Yes	Yes	Yes	Yes	Yes	No	Yes
Biesboer 2021	Yes	No	Yes	Yes	Yes	Yes	Yes	Yes
Berrajaa 2025	Yes	Yes	Yes	Yes	Yes	Yes	No	Yes
Beam 2021	Yes	Yes	Unclear	Yes	Yes	Yes	No	Yes
Barrios 2024	Yes	Yes	Yes	Yes	Unclear	Yes	Yes	Yes
Appiah-Kubi 2024	Yes	Yes	No	Yes	Unclear	Yes	No	Yes
Antoun 2020	Yes	Yes	Yes	Yes	Yes	Yes	No	Yes
Ang 2025	Yes	Yes	Yes	Yes	Yes	Yes	No	Yes
Ali 2020	Yes	Unclear	Yes	Yes	Unclear	Yes	No	Yes
Al-Kourainy 2020	Yes	Yes	No	Yes	Yes	Yes	Yes	Yes
Akkawi 2023	Yes	Yes	Yes	Yes	Yes	Yes	No	Yes
